# Implementing patient reported outcome measures (PROMs) in palliative care - users' cry for help

**DOI:** 10.1186/1477-7525-9-27

**Published:** 2011-04-20

**Authors:** Claudia Bausewein, Steffen T Simon, Hamid Benalia, Julia Downing, Faith N Mwangi-Powell, Barbara A Daveson, Richard Harding, Irene J Higginson

**Affiliations:** 1 King's College London, Cicely Saunders Institute, Department of Palliative Care, Policy and Rehabilitation, London, UK; 2 Deutsche Gesellschaft für Palliativmedizin, Berlin, Germany; 3 Formerly African Palliative Care Association, Kampala, Uganda; 4 African Palliative Care Association, Kampala, Uganda

## Abstract

**Background:**

Patient-reported outcome measurement (PROM) plays an increasingly important role in palliative care. A variety of measures exists and is used in clinical care, audit and research. However, little is known about professionals' views using these measures. The aim of this study is to describe the use and experiences of palliative care professionals with outcome measures.

**Methods:**

A web-based online survey was conducted in Europe and Africa. Professionals working in clinical care, audit and research in palliative care were invited to the survey via national palliative care associations and various databases. Invitation e-mails were sent with a link to the questionnaire.

**Results:**

Overall participation rate 42% (663/1592), overall completion rate 59% (392/663). The majority of respondents were female (63.4%), mean age 46 years (SD 9). 68.1% respondents from Europe and 73.3% from Africa had experiences with outcome measures in palliative care. Non-users reported time constraints, burden, lack of training and guidance as main reasons. In clinical care/audit, assessment of patients' situation, monitoring changes and evaluation of services were main reasons for use. Choice of OMs for research was influenced by validity of the instrument in palliative care and comparability with international literature. Main problems were related to patient characteristics, staff, and outcome measures. Participants expressed the need for more guidance and training in the use of PROMs.

**Conclusions:**

Professionals need more support for the use and implementation of PROMs in clinical practice and research through training and guidance in order to improve patient care.

## Background

Patient-reported outcome measurement plays an increasingly important role in health care in allowing patients to assess the effect and quality of their care [1]. Patient reported outcome measures (PROMs) are used in clinical care (e.g. assessing the health status and needs of patients in a hospital at admission), audit (quality assurance of services) and research (e.g. studying the effectiveness of an intervention). The measurement of effects and outcomes on patients is also central to end-of-life (eol) care and the conduct of research in eol care. Palliative care services should be committed to excellence and high quality of care requiring the regular and systematic evaluation of the processes of care and measurement of outcomes data using validated instruments [2]. In the future, commissioning of services will be based on outcomes rather than activity and PROMS will have a central role in this [3].

Although initiatives were started over the last decade to improve outcome measurement in palliative care [2,4,5] a clear roadmap or uniform approach to measuring outcomes in eol clinical care and research is lacking [2]. In consequence, there are differences in the interpretation of results of studies, meta-analyses are often limited because no core measure is used, and, importantly, some studies fail because they have used inappropriate measures without adequate responsiveness to change. In clinical care, formalised assessments of patient-reported outcomes may increase clinicians' attention to patient concerns which are often overlooked [6]. Recent reports from a clinical workshop indicate that assessment and measurement are important domains, and form a priority area in eol research [7]. Thus, within the midst of an increasing ageing European population, and a continued underspend (0.5%) in eol cancer research [8], a common approach regarding outcome measurement is urgently required.

Professionals play a central role in the utilisation of PROMs, but besides a few studies on professionals' knowledge of quality of life instruments [9-11] little is known about their experiences in the selection and implementation of PROMs in clinical care and research. However, their views can give important insights how outcome measurement could be promoted and harmonised.

PRISMA (**"**Reflecting the **P**ositive dive**R**sities of European pr**I**orities for re**S**earch and **M**easurement in end-of-life c**A**re**") **is a three-year project funded by the European Commission with a focus on co-ordinating outcome measurement in palliative care [12]. One of the aims is to map and harmonise approaches and experiences in eol care measurement. Within PRISMA, one programme of activities concentrates on the experiences of professionals using PROMs in eol care, and on two widely used outcome measures in particular, the Palliative Care Outcome Scale (POS) and the Support Team Assessment Schedule (STAS). The STAS is a tool to evaluate the work of palliative care support teams measuring patient symptoms, anxiety and insight, family anxiety and insight, quality of communication with health care professionals and carers, and need for practical support [13]. The POS consists of ten items assessing physical symptoms, emotional, psychological and spiritual needs, and provision of information and support both from a patient's and professional carer's perspective [14]. Although PRISMA is a European project, one of the members is the African Palliative Care Association (APCA), as research and outcome measurement play an important role in the young history of African palliative care [15].

We report here a study aimed to describe the use and experiences with PROMs of professionals working in palliative care in Europe and Africa. In particular we aimed to describe:

1. reasons of those not using PROMs in palliative care;

2. practice of use (purposes, frequencies etc.) of PROMs in clinical care/audit and research;

3. views of users regarding advantages and challenges of using PROMs;

4. participants' views on further development of PROMs.

Our study responds to the call for harmonising research and best practice across Europe, and in doing so addresses an urgent need for improved measurement in eol care.

## Methods

A web-based online survey was conducted following the Checklist for Reporting Results of Internet E-Survey (CHERRIES) [16]. Survey development included expert review within the PRISMA group, and the electronic version was piloted in seven European countries (Austria, Germany, Italy, Netherlands, Norway, Portugal and UK) with 20 professionals working in palliative care.

The questionnaire included questions on:

• use of tools in general including questions for those not using tools;

• use of tools and PROMs in clinical care/audit and research (purpose, selection, frequency of use, problems and advantages using tools, overall experience);

• further development of tools and resources (characteristics of ideal tool, content of web-based resources etc.);

• respondent information (demographics, profession, setting, country etc.).

Additional open questions gave respondents the opportunity to share their views and experiences.

The survey was conducted in English due to resource constraints. Open questions gave respondents the opportunity to share their views and experiences in their native language. Following a competitive tendering process as per European Commission guidelines, the Centre of Evaluation and Methods (ZEM) at the University of Bonn/Germany http://www.zem.uni-bonn.de was commissioned to conduct the online survey. Special attention was given to a user-friendly outlook, support features during completion of the survey such as alerts for unanswered questions, "back buttons" for reviewing answers and data protection to ensure confidentiality and anonymity. "Adaptive questioning" was used where certain questions were only displayed based on the responses to other items to reduce the number and complexity of the questions. To improve completion rate, only one question was displayed per page on the screen. Overall, the questionnaire contained 59 questions (screens).

A Secure Sockets Layer Virtual Private Network (SSL VPN server) was used to ensure safety and anonymity of participants. Contact details of participants and answers to the survey were saved on two different servers. It was decided not to include a link with a password. Providing passwords would have enabled participants an individualised access to the survey in order to come back to the questionnaire. However, data protection did not allow use of member lists of the national palliative care associations. In these scenarios, the national associations sent the invitation e-mails to around 2000 people.

### Sampling

The sampling frame for the survey were professionals working in palliative care (e.g. doctors, nurses, other professionals) either in clinical care, audit or research in Europe or Africa.

The following institutions and databases were used to sample potential participants in order to reach a maximum of experiences and diversity of professional backgrounds (see Table 1).

**Table 1:** European Palliative Care Associations participating in the online survey

Country	Association		No. of members	Sample
Belgium	FPCF	Federatie Palliatieve Zorg Vlaanderen	About 200 (mixed individuals & organisations)	33%
			
	FWSP	Fédération Wallonne des Soins Palliatifs		

Germany	DGP	Deutsche Gesellschaft für Palliativmedizin e.V.	3,100	20%

Italy	SICP	Società Italiana di Cure Palliative	2,454 (1092 doc, 829 nurse, 375 other = 2,296)	20%

Nether-land	NPTN	Netherlands Palliative Care Network for Terminally Ill Patients	102 (mixed including organisations)	33%

Norway	NFPM	Norwegian Association for Palliative Medicine	112 (only physicians)	33%

Portugal	APCP	Portuguese Association of Palliative Care	406	33%

Spain	SCBCP	Soc. Catalano-Balear de Cures Palliatives	360	33%
	
	SECPAL	Sociedad Española de Cuidados Paliativos	1500	20%

UK	APM	Association for Palliative Medicine of Great Britain & Ireland	1,030 (only physicians)	20%
	
	APCSW	Association of Palliative Care Social Workers	309 (only social workers)	33%
	
	RCN	Royal College of Nursing - Palliative Nursing Group	3,196 (only nurses)	No sampling possible^#^
	
		UK Nurse consultants	28	100%

Other contacts	POS database	Department of Palliative Care, Policy & Rehabilitation, KCL	211	100%
	
	POS & STAS authors	Systematic review of literature	61	100%
	
	Pall care academics in Europe	Chairs & other researchers in palliative medicine in Europe	61	100%
	
	PRISMA members		38	100%

# invitation letter sent to all and request for link to survey demanded

#### 1. National Palliative Care Associations in Europe

Palliative care associations in eight countries of the PRISMA collaboration (Belgium, Germany, Italy, Netherlands, Norway, Portugal, Spain and UK) were invited to participate in the survey. To adjust for the different size of countries and associations, a weighted random sample of 20% was drawn from each group if the total number of clinicians (physicians, nurses and other) in the associations was over 500 members and 33% if the total number was less than 500 members.

#### 2. African Palliative Care Association (APCA)

The African Palliative Care Association (APCA) is a membership organisation for individuals and organisations interested in and working in palliative care in Africa. APCA has 475 individual contacts from 25 African countries. As the list does not contain information on professional background of members, invitation e-mails were sent to all members but those who were not clinicians were asked to feed this information back to the APCA office. This procedure should help to assess the number of non-respondents, taking into account those who are non-clinicians.

#### 3. Other international contacts

The Department of Palliative Care, Policy & Rehabilitation, King's College London's database containing registered POS users was used for the invitations as well as a list of POS and STAS authors who were identified from a systematic review on the use of POS and STAS [17]. Within PRISMA, a list of 61 chairs and researchers in palliative medicine in Europe was established [18]. All contacts of this list were invited to participate in the survey. Finally, all 38 members of the PRISMA consortium were invited to participate in the online survey.

### Data collection

The European online survey was conducted in October and November 2009 with two reminder emails. Invitation e-mails with information about the content and the purpose of the survey, the time for completion, the principal investigator and a link to the web-based survey were sent out to potential participants either by the palliative care associations or directly to named individuals from the POS database, the POS and STAS authors, and European palliative care academics (text of invitation e-mail in Additional file 1). Overall, 2000 e-mails were sent to professionals working in palliative care in Europe. On the first page of the online questionnaire, information about the survey and instructions how to fill in the questionnaire were provided (see Additional file 2).

Due to some delays with ethics approval for the African part of the survey, the African online survey was conducted in January/February 2010 with one reminder. Overall, 487 invitation e-mails were sent out to APCA members initially. Sixty-five emails were returned to the APCA office. For the reminder, 422 e-mails were sent.

To increase participation in the survey we offered a low-cost prize draw to respondents with book tokens for a random number of respondents.

### Analysis

Following the CHERRIES checklist, the participation rate was calculated dividing the number of replies to the first question by the number of unique site visitors (defined as those visiting the first page of the online survey) and the completion rate was calculated by the number of participants answering the first question divided by the number of people submitting the last question [16]. Descriptive analysis of all questions was conducted using frequencies for categorical data and means and SD for continuous variables. Answers from open-ended questions were analysed using content analysis [19]. First, the answer options were collated and then coded looking for emerging themes. We analyzed and report responses for Europe and Africa separately, not to compare responses for these continents, but to give a picture in both regions of the state of science in PROMs and future needs. As the POS database and POS author list included people from all over the world, 17 respondents from the first survey came from outside Europe (Malaysia, Taiwan each n = 1; Australia, Brazil, US, each n = 2; Canada, Japan, Thailand each n = 3). We grouped these participants in two groups (Australia/Canada/Japan/US (ACJU), n = 10; Brazil/Malaysia/Taiwan/Thailand (BMTT), n = 7) in relation to their health care system and socio-economic status. As the data from Europe and ACJU and also the data from Africa and BMTT were similar regarding the demographic variables we merged these groups accordingly. SPSS Statistics version 17.0 was used for quantitative and NViVo 8 to assist in qualitative data analysis.

### Ethics

Ethic approval was obtained from the Research Ethics Committee at King's College London (BDM/08/09-102). We were advised that further ethics approval from all other countries was not needed as this survey did not include patients but only professionals. For Africa, ethics approval was obtained from the Ethics Committee of the Ugandan National Council for Science and Technology (IS 62).

## Results

1592 unique site visitors (1291 in Europe and 301 in Africa) were counted on the first page of the survey (which gave more specific information about the survey). The overall participation rate was 42% with 663/1592 professionals answering the first question and 392/663 completing the last question (completion rate 59%). In Europe the participation rate was 38% (495/1291) and the completion rate 63% (311/495), whereas in Africa the participation rate was 56% (168/301) and the completion rate 48% (81/168).

### Sample characteristics

Demographic data was available from 379/663 participants. 63.4% of respondents were female; mean age of all respondents was 46 years (SD 9). Both in Europe and in Africa, the majority had a clinical background, about 10% were researchers and about 19% of respondents in Europe and 26% in Africa had both a clinical and a research background (see Table 2). In both continents, about two thirds of participants had more than five years experience in palliative care.

**Table 2:** Characteristics of respondents

	Europe n (%)	Africa n (%)
**Background of respondents**	**n = 291**	**n = 88**

Clinician (doctor, nurse, social worker, therapist, etc.)	211 (72.5%)	55 (62.5%)

Researcher	26 (8.9%)	10 (11.4%)

Both	54 (18.6%)	23 (26.1%)

**Experience in palliative care**	**n = 291**	**n = 87**

≤ 5 years	101 (34.7%)	32 (36.8%)

6 - 10 years	81 (27.8%)	21 (24.1%)

> 10 years	108 (37.1)	33 (37.9%)

**Background of clinicians**	**n = 266**	**n = 78**

Medicine	160 (60.2%)	31 (39.7%)

Nursing	68 (25.6%)	32 (41.0%)

Other profession	38 (14.3%)	15 (19.2%)

**Background of researchers**	**n = 80**	**n = 33**

Medicine	46 (57.5%)	8 (24.2%)

Nursing	10 (12.5%)	8 (24.2%)

Psychology	8 (10.0%)	3 (9.1%)

Social Science	5 (6.3%)	8 (24.2%)

Other profession	11 (13.8%)	6 (18.2%)

In Europe, physicians constituted the largest group both of clinicians and researchers. Every fourth clinical respondent and every eighth researcher had a nursing background. In Africa, nurses were the largest group of clinicians but medicine, nursing and social science were the most common backgrounds of researchers. About 30% of researchers in Europe and over 50% in Africa had a background other than medicine or nursing, such as psychology, social science, or the health sciences. 78% of respondents in Europe and 64% in Africa came from a specialist palliative care background.

Participants came from a variety of European countries with 37% from the UK, 15% from Italy, 12% from Germany, and 7% each from Spain and Portugal. In Africa, a third of respondents originated from Uganda and about 20% came from Kenya. Further responses came from 20 other African countries. About 6% participants replied from countries outside of Europe or Africa (e.g. Australia, Canada, Thailand, US, Brazil). Their data has been merged into the European and African data set as described in the methods section.

#### General use of PROMs in palliative care

**Figure 1:**
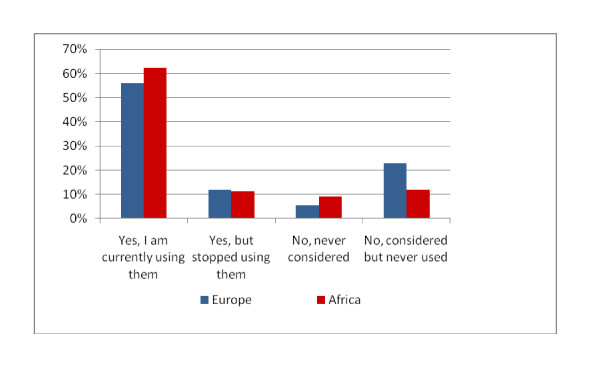
**Use of outcome measures in palliative care**.

The majority of respondents in Europe (68.1%) and in Africa (73.6%) had experiences with PROMs in palliative care and more than half of respondents in both continents were using PROMs at the time of answering the survey (see Figure 1).

Main reasons for *not *using PROMs differed slightly between Europe and Africa (see Table 3). In Europe, time constraints and patient factors were the main reasons for not using PROMs (each about 25%) but lack of training (20%) and guidance (17%) were mentioned frequently as well. These reasons were mentioned even more often by African respondents whereas patient related factors such as tools are too burdensome or do not reflect patients' situation played a minor role in the views of African professionals.

**Table 3:** Reasons for not using outcome measures and circumstances starting using them (multiple answers possible)

	Europe n (%)	Africa n (%)
**Main reasons for not using outcome measures**	**n = 201**	**n = 63**

Time constraints	52 (25.9%)	11 (17.5%)

Tools are too burdensome for patients & families	47 (23.4%)	2 (3.2%)

Lack of training about how to use tools	41 (20.4%)	13 (20.6%)

Tools do not reflect the patient's situation	39 (19.4%)	2 (3.2%)

Tools are too burdensome for staff	36 (17.9%)	3 (4.8%)

Lack of guidance about how to use tools	35 (17.4%)	15 (23.8%)

Don't know where to get information about tools	31 (15.4%)	8 (12.7%)

Lack of training about how to analyse data from tools	30 (14.9%)	12 (19.0%)

Not enough staff	30 (14.9%)	7 (11.1%)

Limited access (e.g. registration needed)	13 (6.5%)	2 (3.2%)

Language restriction (e.g. tools not translated)	12 (6.0%)	6 (9.5%)

Cost constraints (e.g. fees for tools)	7 (3.5%)	4 (6.3%)

Lack of validated version for specific setting (e.g. Africa)	4 (2.0%)	10 (15.9%)

**Circumstances to start use of outcome measures**	**n = 192**	**n = 52**

If more information and guidance were provided about tools	86 (44.8%)	26 (50.0%)

If more training was provided	58 (30.2%)	20 (38.5%)

If I had more time	56 (29.2%)	10 (19.2%)

If appropriate tools were available (e.g. translations)	42 (21.9%)	22 (42.3%)

If I had more staff	42 (21.9%)	7 (13.5%)

I don't think I would ever use tools	16 (8.3%)	0 (.0%)

If I had more money	9 (4.7%)	6 (11.5%)

Asked under which circumstances respondents would start using PROMs, the provision of information, guidance and training were named most often in both continents (Table 3). Only a minority in Europe and nobody in Africa thought that they would never use PROMs in palliative care. Overall, in Europe time, burden, and the feeling that measures don't reflect patients' situation played a bigger role compared to African respondents who mainly described the lack of available measures and translations.

#### Use of PROMs in clinical care and audit

A variety of purposes for the use of PROMs in clinical care/audit were named by respondents. Main reasons for the majority in Europe and in Africa were assessment of the patients' situation, monitoring changes in patients' health status and evaluating the effect of an intervention. The latter, assessing families' needs and use in audit played a bigger role in Africa compared to Europe (see Table 4).

**Table 4:** Purpose and advantages using outcome measures in clinical care/audit (multiple answers possible)

	Europe n (%)	Africa n (%)
**Purpose of using outcome measures in clinical care/audit**	**n = 254**	**n = 86**

To assess patients' symptoms/needs/problems	234 (92.1%)	77 (89.5%)

To monitor changes in patients' health status or quality of life	180 (70.9%)	58 (67.4%)

To evaluate the effect of an intervention/care/service	172 (67.7%)	68 (79.1%)

To facilitate communication in the team	117 (46.1%)	35 (40.7%)

To document patients' characteristics	116 (45.7%)	37 (43.0%)

To assess families' needs/problems	111 (43.7%)	51 (59.3%)

To facilitate communication with patients/families	85 (33.5%)	40 (46.5%)

To assess the care given against standards (in audit)	59 (23.2%)	45 (52.3%)

Other, please state	9 (3.5%)	12 (14.0%)

**Advantages of using outcome measures**	**n = 233**	**n = 78**

To better understand patients' and families' needs (e.g. assessment, screening)	186 (79.8%)	72 (92.3%)

To improve the quality of care for patients and families	172 (73.8%)	72 (92.3%)

To make decisions regarding treatment/care	170 (73.0%)	73 (93.6%)

To communicate in the team	147 (63.1%)	56 (71.8%)

To evaluate services	121 (51.9%)	64 (82.1%)

To communicate with the patients and families	102 (43.8%)	58 (74.4%)

Other, please state	16 (6.9%)	14 (17.9%)

Advantages of using PROMs were better understanding of patients' and families' needs, improvement of quality of care and help in decision making.

76% of results of outcome measurement were discussed in clinical meetings, 74% were documented in patients' records and 73% were used to inform or determine treatment or care. Less than half of the respondents used results for discussions with patients or families.

The overall experience of using PROMs in clinical care and audit was rated as good or very good by 70% of respondents in Europe and 76.4% in Africa. Only around 7% in both continents reported bad or very bad experiences.

#### Use of PROMs in palliative care research

The majority of those using PROMs in research in Europe and in Africa used it to measure patients' symptoms or quality of life and to evaluate the effect of an intervention (Table 5). Measurement of quality of care seemed to be more important in Africa than in Europe.

**Table 5:** Purposes for using outcome measures and factors influencing the choice of an outcome measure in research (multiple answers possible)

	Europe n (%)	Africa n (%)
**Purpose of using outcome measures in research**	**n = 119**	**n = 48**

To measure/describe patients' symptoms	90 (75.6%)	35 (72.9%)

To measure/describe patients' quality of life	77 (64.7%)	40 (83.3%)

To evaluate the effect of an intervention/care/service	77 (64.7%)	36 (79.2%)

To assess patients' functional status	71 (59.7%)	29 (60.4%)

To monitor changes in patients health status or quality of life	57 (47.9%)	32 (66.7%)

To measure/describe patients' quality of care	44 (37.0%)	33 (68.8%)

To screen whether patients meet inclusion criteria	37 (31.1%)	15 (31.3%)

**Factors influencing choice of outcome measures in research**	**n = 118**	**n = 46**

Validated in palliative care	80 (67.8%)	34 (73.9%)

Comparability with national and/or international literature	76 (64.4%)	20 (43.5%)

Tool previously used in similar setting/patient group	70 (59.3%)	24(52.2%)

Time needed for completion	64 (54.2%)	26 (56.5%)

Existing translation for my language/country	63 (53.4%)	18 (39.1%)

Access to tool	59 (50.0%)	30 (65.2%)

Validated in patient group (e.g. disease)	59 (50.0%)	20 (43.5%)

Own previous experience with a tool	53 (44.9%)	15 (32.6%)

Cost (e.g. fees to use tool)	23 (19.5%)	16 (34.8%)

Validation of a tool in palliative care population was the main factor that influenced researchers' choice of an outcome measure in both continents. Access to a tool and time needed for completion were additional important factors in Africa. In Europe, comparability with the national and international literature and previous use of the tool in a similar setting or patient group were further factors that influence the choice of a tool (see Table 5).

Overall experiences using PROMs in research were judged as good or very good by almost two thirds of researchers in Europe (64.7%) and more than four in five respondents in Africa (82.5%). About a third in Europe and 15% in Africa judged their experiences as neither good nor bad. Only a minority of 2% reported bad experiences.

#### Problems and barriers using PROMs in palliative care

In the open questions, participants expressed a variety of problems and barriers for the use of PROMs in clinical care, audit and research (see Table 6). They can be related to patients, staff and the PROMs itself. Patients in palliative care are described as a vulnerable group, often too ill to fill in PROMs due to the disease or cognitive impairment, or find it difficult to understand what is wanted of them. Staff barriers relate to gate keeping, lack of time, lack of training or general reluctance to use PROMs in palliative care. Issues that were mentioned related to PROMs were access to and choice of tools, questions around validity and reliability, adaptation in different languages and cultures, and complexity of the measures. In addition to the described areas, researchers reported specifically problems around data analyses and dealing with missing data.

**Table 6:** Views of respondents on the use of PROMs in palliative care

Positive experiences using tools
• "acceptance of the experience by staff in spite of initial reluctance"
• "good information to share with staff as affirmation of care and in identifying areas of improvement"
• "the tools turned out to be exceptionally helpful referring to our negotiations with commissioners"

**Problems and barriers using tools**

• "staff sometimes take the audits as criticism and not as constructive criticism"
• "some health professionals are not sure how to use them (PROMs) or understand the benefit of them and so consistency can be a problem - particularly if trying to look at change over time and if a different person is asking on different occasions. Sometimes the health professionals complete the tool but do not look at what it is telling them and use this to help the patient - they are just ticking the box to say that they have used them"
• "scores don't always correspond to how the patient is feeling"
• "some patient groups have more difficulty than others completing them e.g. hospital inpatients achieved 50% response rate whenever used patient completed tool"

**Development of web-based resources**

• "updated info in regard to symptoms management, spiritual care, psychosocial interventions, including case-studies when available"
• "recommended reading resources (research etc.)"
• "training opportunities for multi-disciplinary staff members"
• "list of centres using the tool for exchange of experience (or even creating control groups in terms of research)"
• "comparative information on different tools to ease choice (as far as possibly in lay-friendly terms); access when possible to PDF or word document of the tool; contact details and requirements of use (e.g. payment); link to validation paper and other publications; accounts of researchers who used the tool"

**Future development of PROMs in palliative care**

• "don't invent another one! Use/develop the ones we currently have"
• "I think there is enough already and we should focus on identifying the best and then working out how to implement/integrate them in routine care"
• "if there can be training on how to use the tools it would be better because it is important"
• "keep them easy, otherwise implementation will be difficult. Tools are only as good as the people who use them, and I often prefer reading through someone's narrative account of a situation rather than looking at numbers"

#### Future development of PROMs in palliative care

Respondents also had the opportunity to comment on further development of PROMs in palliative care (see see Table 6). It emerged that people would prefer simple tools relevant to clinical practice. During the development of such tools the service-user perspective needs to be taken more into account. Respondents favoured tools in an electronic format with the possibilities to choose from different modules according to patient situation and condition. Many respondents commented that there were an ample number of OMs and that our focus should be on the improvement of existing measures rather than the development of new tools.

#### Development of web-based resources

Answers on development of web resources for the use of PROMs highlighted the need to provide interactive content with clinical cases and training examples in addition to detailed information about specific PROMs and related references (see see Table 6). The need for guidance on how to use an outcome measure and analyse data was expressed. Professionals wish to communicate with each other using comments, fora, and sharing content (i.e. advice, syntax for analysis). This need for communication and sharing information is not met by current websites focussing on outcome measurement in palliative care.

## Discussion

This is the first international survey conducted in Europe and in Africa seeking the views of professionals on PROMs in palliative care, their experiences with PROMs in clinical care, audit and research, and their perceived need regarding future development of measures and necessary resources.

About a third of respondents in Europe and about a fifth of those in Africa reported not using PROMs in palliative care for several reasons. Only a few respondents precluded that they would ever use PROMs. This suggests a general openness for PROMs even of those respondents who don't currently use them. However, it might be argued that only people with a positive attitude towards PROMs participated in this survey.

PROMs help professionals in a variety of ways, including better understanding patients' and families' situations, monitoring change and evaluating interventions. Choice of PROMs is hindered by the huge numbers of tools available with many of them not being developed for the clinical but rather for the research setting [20]. In research, a variety of factors influence choice of an outcome measure but validation of the PROM in palliative care was most important for European and African researchers. Comparability with national and/or international literature plays a bigger role in Europe whereas access to tools is an important issue in Africa.

Only few studies assessed professionals' views on outcome measurement [10,11,21]. Young and Maher identified resource issues, missing data, communication skills and site of questionnaire completion as main problems of collecting quality of life data in EORTC clinical trials [10]. Oncologists appeared to be willing to use quality of life measures but expressed a more positive attitude and more willingness to use quality of life data than what their reported current behaviour indicated that they are actually doing [11].

The lack of training and guidance was one of the main reasons mentioned by those not using PROMs but also emphasized by those regularly using PROMs. If outcome measurement should become routine in clinical palliative care as has been proposed [8], the development of guidance how to use PROMs and of resources for learning and teaching seems to be crucial to support professionals in this area. Guidance should also provide support to overcome barriers such as gate keeping, burden for patients and access to PROMs. Projects such as the Palliative Care Outcomes Collaboration in Australia which is a national approach towards the routine assessment of palliative care using standardised assessment tools, is already providing training for professionals [22]. Thus, development of guidance and educational material will be an important step to improve professionals' knowledge on outcome measurement and is part of the work of the PRISMA collaborative [12]. Increased knowledge is important but has been challenged as not sufficient enough to change behaviour [23]. Thus, such materials have to be embedded in multiple strategies to overcome different barriers [24]. Another strategy could be to develop competency-based training programs which are learner- or participant-centered taking into account the individual situation and the clinical environment of the professional [25].

Institutional barriers such as time constraints, and gate keeping were more prominent in Europe than in Africa. Both factors are not necessarily palliative care specific although gate keeping is known to be an important factor for professionals not recruiting patients to palliative care research [9]. This survey shows in addition that it is also a barrier for the regular use of PROMs in palliative care. Time constraints could be addressed by training on outcome measurement as it might help to reduce time that it takes to complete measures.

To aid further development of outcome measurement in palliative care, international and national palliative care and professional organisations should be mobilized and encouraged to collaborate similar to successful collaborations in oncology, such as the EORTC, the ECOG, or the interRAI collaborative in geriatrics [26].

### Strengths and limitations

This survey included professionals from two major regions, Europe and Africa. Palliative care has developed over the last decade in Africa in a more structured way [15], and our survey enabled access to the workforce on this continent. The web-based design allowed us to reach more professionals than would have been feasible with a postal survey, both in Europe and in Africa. This survey also represents a wide range of professionals from a variety of medical, nursing and other backgrounds reflecting the multiprofessional and interdisciplinary work in palliative care. This reflects daily practice as PROMs are used in a variety of ways and by various professionals. Particularly the views of clinicians are not often heard in outcome measurement.

There are some limitations to this online survey. First, the results of this survey may be biased as only professionals with a positive attitude towards PROMs may have participated in the survey. Overall experiences of clinicians and researchers with PROMS were similarly positive in Europe and in Africa and only a minority of respondents in both groups had negative experiences. However, about a third of European respondents and about a quarter of African professionals stated that they were not using PROMs or have stopped using them and gave their views on this. Thus, we were able to collect a range of views.

Second, invitations to participate in the survey were only sent out via email and not by post. This introduces bias towards those professionals having access to the internet. We assumed that the majority of palliative care professionals have internet access at least at work as online communication is now part of everyday life and even more so in medicine. However, we acknowledge that web access may be more problematic in Africa compared to Europe. Therefore, the African sample could potentially be biased and not fully representative of those working in palliative care in Africa. A. As mentioned above, a postal survey would also have had limitations and probably reached less participants.

Third, due to limited resources and limited time, the survey was conducted in English only. The language restriction may be one reason for the low responses from European countries other than the UK where about a third of respondents came from. Similarly, in Africa French speaking countries are potentially underrepresented in this survey due to the language restriction. Nevertheless, the survey represents views from professionals in southern, central and northern parts of Europe, and almost 20 African countries.

Fourth, participation and completion rate (42 and 59%, respectively) could have been higher. We sampled professionals via national bodies to participate in the survey without knowing their actual involvement in palliative care. Non-responders might have been an unknown proportion of professionals without direct experience of palliative care provision or opposing outcome measurement in palliative care for reasons such as patient or staff burden or lack of resources. We captured some of these views in our survey but they might be under-represented. In addition, web-based surveys have known disadvantages with regard to sampling, length of questionnaires and access[27]. We tried to increase the response to the survey sending two reminders and offered a low-cost prize draw with book tokens. We also paid special attention to the user friendliness of the online questionnaire and piloted it with a group of professionals.

It is also important to consider that through sending out invitation e-mails with a link to the survey, no information on individual response status could be collected [28]. And although we tried to contact as many different palliative care associations as possible the distribution of different professions is not as equal as hoped, with a dominance of physicians and nurses, in addition response rate within various regions varied. As the number of online surveys may increase in the future, this is an area of relevance for profession-focussed palliative care organisations.

## Conclusions

Our study answered a call for harmonising research and best practice across Europe, and in doing so we have responded to an urgent need for improved measurement in eol care. Our findings indicate that these respondents, while mostly committed to measuring outcomes, clearly indicated a cry for help in relation to guidance and training in outcome measurement in palliative care. It is concluded that a coordinated response is now required to develop outcome measurement in eol care. Our respondents have provided directions in terms of how this needs to be achieved, including a need for training, guidance, clinically relevant tool refinement. This could comprise mobilising national and international palliative care and professional organisations to optimise the outcomes of the delivery of eol care ensuring that this service is both delivered and measured.

## Competing interests

The authors declare that they have no competing interests.

## Authors' contributions

CB, STS, RH and IJH conceived and designed the study; CB, STS, JD, FMP, BD and IJH developed the online survey; HB, CB, BD and STS analysed the data; CB drafted the manuscript; STS, BD, IJH, RH critically revised the manuscript. All authors read and approved the final document.

## Supplementary Material

Additional file 1 **Survey invitation letter**. Invitation letter sent via e-mail to members of palliative care associations and international contacts with information about the PRISMA project and the online survey, and a direct link to the survey. Click here for file

Additional file 2 **Survey participant information**. First screen of online survey with more detailed information about the online survey. Click here for file
