# Supplementary material for: Implementing patient reported outcome measures (PROMs) in palliative care - users' cry for help
Source: Health Qual Life Outcomes. 2011 Apr 20;9:27. doi: 10.1186/1477-7525-9-27 (PMC3112059; doi:10.1186/1477-7525-9-27)
Supplement: Additional file 1 — Survey invitation letter. Invitation letter sent via e-mail to members of palliative care associations and international contacts with information about the PRISMA project and the online survey, and a direct link to the survey. [file 1477-7525-9-27-S1.PDF]

Dear colleague,

We would like to invite you to participate in a short online survey on measurement tools in palliative care. You have been selected to participate as a member of your national palliative care association.

Your experience is very valuable to us! In palliative care, using tools plays an important role but can be challenging. Please help us by completing this survey.

You have the chance to win a prize! Ten of the first 100 respondents will receive an Amazon voucher worth €50/£40!

The aim of the survey is to inform the development of future resources for outcome measurement. It will take approximately 15 min. to complete.

The survey forms part of PRISMA: *Reflecting the Positive diveRsities of European prIorities for reSearch and Measurement in end-of-life cAre* (Health-F2-2008-201655). PRISMA is a European Commission funded project that aims to inform best practice and harmonise research in end of life care in Europe and Africa. It has been developed by the German Association for Palliative Medicine and the Department of Palliative Care, Policy & Rehabilitation, King's College London. All answers will be handled confidentially.

Thank you for considering completing this questionnaire which will help us to improve the use of measurement tools in palliative care! The survey will end on 31st October.

Please click the following link when you want to start.

http://www.umfragen.uni-bonn.de/mrIWeb/mrIWeb.dll?I.Project=PRISMA

If you have any questions about this survey please contact:

Dr Claudia Bausewein PhD MD MSc

Department of Palliative Care, Policy & Rehabilitation
King's College London
Tel. +44 20 7848 0753
Email: prismawp4@kcl.ac.uk
