# Supplementary material for: Implementing patient reported outcome measures (PROMs) in palliative care - users' cry for help
Source: Health Qual Life Outcomes. 2011 Apr 20;9:27. doi: 10.1186/1477-7525-9-27 (PMC3112059; doi:10.1186/1477-7525-9-27)
Supplement: Additional file 2 — Survey participant information. First screen of online survey with more detailed information about the online survey. [file 1477-7525-9-27-S2.PDF]

0%

Use and experience with measurement tools in palliative care

Dear participant,

Thank you for your interest in this PRISMA survey on 'Use and experiences with measurement tools in palliative care' in Europe and Africa. **Your knowledge and experience is invaluable to us.**

Your feedback will directly assist the development of future resources for outcome measurement in palliative care. There are many terms used for outcome measures such as “instruments”, “measurement tools”, “measures”, etc; **we use the term "tools" in this survey to refer to all such items and to enhance clarity.**

The main focus of the survey is on tools that assess patients’ needs, quality of life, quality of care, and symptoms in adult patients. We are interested in assessment tools that cover multiple symptoms, rather than those that focus on one symptom alone.

All responses to this survey will be handled **confidentially**. Nevertheless, you are invited to give us your contact details separately at the end of the questionnaire, as we plan to invite respondents to an international workshop in Spring 2010 to discuss issues around measurement in palliative care. The results from the survey will also be published in an international journal and will play a key role in the development of future resources for outcome measurement.

Ten of the first 100 respondents will receive an Amazon voucher worth €50 / £40!

The survey is very easy to complete:

- It will take approximately 15 minutes.
- We would be very grateful if you could complete the questionnaire until the **31st October**.
- If you do not want to answer a question immediately, you can simply move on to the next one and return to it later.
- Use the "Previous" and "Next" buttons to navigate through the survey. **Please do not use the navigation buttons in your browser.**
- Along with certain items we provide an additional explanation for you assistance. These items are marked with a hyperlink and an "Info"-Icon . This additional piece of information is shown if you move the cursor over the marked items (Javascript has to be activated).

Please use the "Next" button to begin.

Next

Imprint

If you have any questions about this survey please contact

Dr Claudia Bausewein PhD MD MSc
Dr Steffen Simon MD MSc
Department of Palliative Care, Policy and Rehabilitation
King’s College London

Tel. +44 20 7848 0753
Email: prismawp4@kcl.ac.uk

Help
